# Latent Variables in Cognitive Decline: Caregiver Burden and Behavioral and Psychological Symptoms of Dementia

**DOI:** 10.1093/geroni/igaf122.3184

**Published:** 2025-12-31

**Authors:** Lauren Chrzanowski, Landon Peeples, Benjamin Mast

**Affiliations:** University of Louisville, Louisville, Kentucky, United States; University of Louisville, Louisville, Kentucky, United States; University of Louisville, Louisville, Kentucky, United States

## Abstract

Latent variable modeling has been used to model dementia by examining the covariance between cognitive decline (g) and daily function (f – decline in ability to perform instrumental activities of daily living) as a common factor (gf). The current study uses confirmatory factor analyses (CFA) to replicate this model and expand it to include behavioral and psychological symptoms (p) and family caregiver burden (b) as a second common factor (pb). Participant data (N = 915; older adults without cognitive impairment, with mild cognitive impairment, or with Alzheimer’s disease) was obtained from the Alzheimer’s Disease Neuroimaging Initiative (ADNI) wave 4. To replicate the gf factor, scores from the Montreal Cognitive Assessment (MoCA), logical memory, delayed recall, and Functional Activities Questionnaire (FAQ) were fit in the first model. CFA results supported a 4-variable structure of the neurological factor with acceptable global fit. The second model included the pb factor comprised of Neuropsychiatric Inventory (NPI) subscales (caregiver burden/distress, behavioral and psychological symptoms of dementia (BPSD) frequency, and BPSD severity) and Geriatric Depression Scale scores. The global fit of this second model was excellent (CFI = .998, RMSEA = 0.054). The two-factor model had better fit than a single factor model (CFI = .609, RMSEA = .368), suggesting gf and pb are distinct factors reflecting two aspects of dementia: cognitive/functional decline and behavior change/caregiver burden. These results replicate prior literature and highlight a latent interpersonal and distress variable, which may be experienced by individuals and families impacted by dementia.

